# Dual function and associated costs of a highly exaggerated trait in a cichlid fish

**DOI:** 10.1002/ece3.8383

**Published:** 2021-11-23

**Authors:** Sina J. Rometsch, Julián Torres‐Dowdall, Gonzalo Machado‐Schiaffino, Nidal Karagic, Axel Meyer

**Affiliations:** ^1^ Chair in Zoology and Evolutionary Biology Department of Biology University of Konstanz Konstanz Germany; ^2^ Present address: Department of Functional Biology University of Oviedo Oviedo Spain

**Keywords:** *Amphilophus citrinellus*, armament, dual signaling, Midas cichlids, ornament, secondary sexual characteristic, sexual selection

## Abstract

Exaggerated secondary sexual characteristics are apparently costly and seem to defy natural selection. This conundrum promoted the theory of sexual selection. Accordingly, exaggerated secondary sexual characteristics might be ornaments on which female choice is based and/or armaments used during male–male competition. Males of many cichlid fish species, including the adaptive radiation of Nicaraguan Midas cichlids, develop a highly exaggerated nuchal hump, which is thought to be a sexually selected trait. To test this hypothesis, we conducted a series of behavioral assays in F2 hybrids obtained from crossing a species with a relatively small hump and one with an exaggerated hump. Mate‐choice experiments showed a clear female preference for males with large humps. In an open‐choice experiment with limited territories, couples including large humped males were more successful in acquiring these territories. Therefore, nuchal humps appear to serve dual functions as an ornament for attracting mates and as an armament for direct contest with rivals. Although being beneficial in terms of sexual selection, this trait also imposes fitness costs on males possessing disproportionally large nuchal humps since they exhibit decreased endurance and increased energetic costs when swimming. We conclude that these costs illustrate trade‐offs associated with large hump size between sexual and natural selection, which causes the latter to limit further exaggeration of this spectacular male trait.

## Graphical abstract

Exaggerated secondary sexual characteristics might be ornaments on which female choice is based and/or armaments used during male–male competition, but at the same time, such traits are often costly. Here, we find that exaggerated head humps in Midas cichlids confer dual functions as an ornament for attracting mates and as an armament for contest with rivals, while also imposing fitness costs regarding swimming performance. We conclude that these costs illustrate trade‐offs associated with large hump size between sexual and natural selection, which causes the latter to limit further exaggeration of this spectacular male trait.

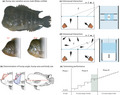

## 1 INTRODUCTION

The evolution of exaggerated secondary sexual characteristics has interested scientists since Charles Darwin (Andersson, 1994; Berglund et al., 1996; Darwin, 1871; Fisher, 1930; Zahavi, 1975). Striking examples of such traits, which are most often sexually dimorphic, can be found in many animals including the extraordinarily long tail feathers of widowbirds (Andersson, 1982a), the wheel of peacocks (Petrie & Williams, 1993), the antlers of red deer (Malo et al., 2005), the horns of some beetles (Emlen et al., 2006), and the colorful face markings in mandrills (Setchell et al., 2006).

Exaggerated traits have two potential functions: they can act as ornaments in intersexual selection and/or as armaments in intrasexual competition (Darwin, 1871). In both cases, they play important roles, for example, in signaling their bearers’ condition to conspecifics of the same or opposite sex (Andersson, 1986; O'Brien et al., 2017; Searcy & Nowicki, 2005; Smith & Harper, 2003; Warren et al., 2013). Ornaments are beneficial during intersexual encounters and are supposed to increase the sexual attractiveness of their bearers (Andersson, 1994; Berglund et al., 1996). Females use differences in the expression of exaggerated male ornaments, for example, tail length in swordtail fish (Meyer, 1997), to evaluate and choose among potential mates. In contrast, armaments (*sensu* Berglund et al. (1996), i.e., weapons and status signals) are typically important in direct male–male competition and have been suggested to evolve through intrasexual selection (Andersson, 1994; Berglund et al., 1996; O'Brien et al., 2019; Rico‐Guevara & Hurme, 2019). Size of armaments, for example, antler size in deer (Lincoln, 1972), helps males assess dominance status and fighting ability of rivals. Additionally, some exaggerated traits have dual signaling functions (reviewed in Berglund et al., 1996). For instance, carotenoid feather signals in rock sparrows represent both ornaments and armaments (Griggio et al., 2007).

Due to associated benefits in reproductive success, directional sexual selection is assumed to drive the evolution of secondary sexual traits toward exaggeration. Continuous trait increase will be checked by natural selection since, at some point, the associated benefits will be surpassed by the costs imposed by bearing the trait (Andersson, 1982b). Therefore, a trade‐off between the advantages conferred by sexual selection and the disadvantages imposed by natural selection appears to shape the phenotype of these traits (e.g., Allen & Levinton, 2007; Clark & Dudley, 2009; O'Brien et al., 2019). For example, in hummingbirds, elongated male tails are not only preferred by females but also increase metabolic costs during flight performance (Clark & Dudley, 2009). Therefore, the evolution of exaggerated sexually selected signals can be hindered by natural selection when traits become too costly.

**FIGURE 1:**
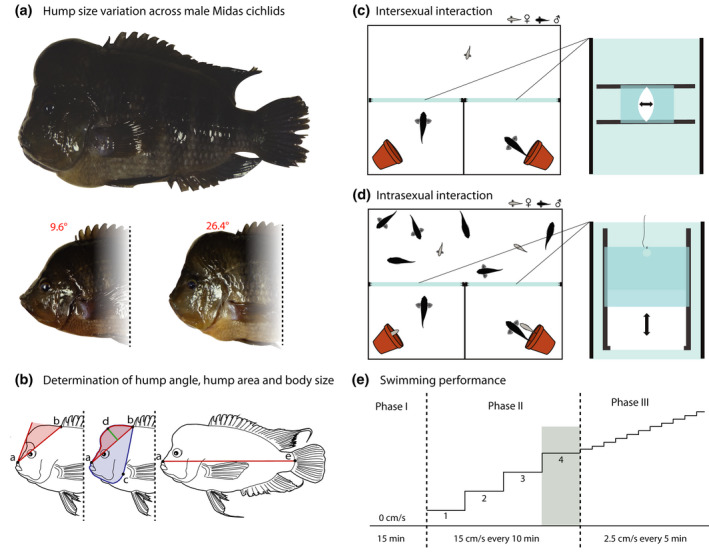
Overview of experimental procedures conducted throughout this study. (a) Nuchal humps can be very pronounced in Midas cichlids. However, their size can vary considerably among males. (b) Lateral view of a Midas cichlid. Left: hump angle resulting from the intersection of two lines at the top of the upper lip (*a*). The first line is located between *point* *b* (insertion of the dorsal fin) and *point* *a*, while the second line is the tangent to the protruded forehead starting from *point a*. Middle: corrected hump area is defined as the ratio of the hump area (indicated by the area in red) to the head area (indicated by the area in blue). The hump area starts from *point b*, follows the shape of the fish's head to *point a* and is then completed by a line between *point a* and *b*. The head area covers the area starting from *point b*, following the shape of the fish's head to *point c* (operculum) and is completed by a line drawn from *point c* to *b*. Large values for the total hump area indicate big humps, while small values are obtained from fish with small humps. Corrected maximum hump height is defined as the ratio of the maximum hump height (*point d*, indicated by the green line) to standard body length (described on the right). The maximum hump height is the maximum height of the hump perpendicular to the line between *point a* and *b*). Right: distance between *points a* and *e* indicates standard body length. (c) Left: experimental setup was divided into three compartments: one large and two small ones. One male was confined in each of the small compartments and only the female could enter and leave all three compartments. Right: A convex gate could be adjusted to the female's size to ensure selective passage of only the female between the different compartments. (d) Left: experimental setup was divided into three compartments: one large and two small ones. Flowerpots in each small compartment provided shelter for breeding couples. Right: upon couple formation, a gate could be released to trap the pair inside the compartment and therefore facilitate their removal from the experiment. (e) Determination of swimming performance: following a 15‐min acclimation period, velocity was increased in 10‐min time intervals by 15 cm s^−1^ increments. Over the first 93 s of the 60 cm s^−1^ increment (indicated in green), pectoral fin beats were counted. After the end of the 60 cm s^−1^ increment, flow velocity was increased every 5 min by 2.5 cm s^−1^ increments until the fish reached exhaustion

Exaggerated traits thought to evolve through sexual selection are found throughout the entire fish family Cichlidae and play important roles in inter‐ and intrasexual communication (Anderson et al., 2016; Maan et al., 2004; Seehausen & van Alphen, 1998; Taylor et al., 1998). The Nicaraguan Midas cichlid species complex (*Amphilophus* spp.) is an extremely young radiation (i.e., about 16,700 years old; Barluenga & Meyer, 2004; Barluenga et al., 2006; Kautt et al., 2016; Kautt et al., 2018; Kautt et al., 2020) and exhibits multiple interesting traits such as body coloration (color polymorphism; Henning et al., 2013), hypertrophied lips (Machado‐Schiaffino et al., 2014), and body shape (parallel evolution of body shape along a benthic‐limnetic axis; Elmer et al., 2014). One particularly extravagant feature is the exaggerated swelling of the fish's forehead, referred to as a nuchal hump, which results in a peculiar head shape (Figure 1a; Barlow & Ballin, 1976; Lecaudey et al., 2019).

Nuchal humps are known to be closely associated with the reproductive cycle in members of the genus *Amphilophus* (Barlow, 1976). For the most part, non‐breeding individuals are isomorphic except for variation in the hump size which gets further exaggerated in males when ready to mate (Barlow & Ballin, 1976; McKaye, 1986). Pair formation in the monogamous Midas cichlids is preceded by a thorough quality assessment of potential mates, since their elaborate parental care involves both parents’ defense of offspring for weeks (Barlow, 1992; Rogers, 1988). As territory loss is frequent, Midas cichlids are highly aggressive toward intruders (Holder et al., 1991; McKaye, 1977).

To date, competing hypotheses for the evolution and function of exaggerated nuchal humps in cichlids have been proposed, including their evolution to store fat (Barlow, 1998) or to facilitate sex recognition (Barlow & Siri, 1997). However, these hypotheses do not provide sufficient or satisfactory explanations given that the amount of adipose tissue is not altered during nuchal hump development (Bleick, 1975), and sex recognition is facilitated not only by a clear size dimorphism exhibited by males and females (Francis & Barlow, 1993) but also by a potentially important role of chemical communication between the sexes (reviewed in Rometsch et al., 2020). In this study, we explored the role of nuchal humps in male F2 hybrid Midas cichlids in mating success as ornaments and/or armaments, through a combination of limited‐choice and open‐choice experiments, as well as the potential associated swimming costs of nuchal humps. First, we investigated the role of exaggerated nuchal humps as ornament by determining whether there is a female preference for males with large humps. Next, we determined whether nuchal humps function as armaments, which might signal male quality, when male Midas cichlids compete for breeding territories. Finally, we explored whether nuchal humps impose a cost in terms of natural selection, specifically swimming performance, that might explain how further exaggeration of this trait is limited.

## 2 MATERIALS AND METHODS

### 2.1 Species selection and maintenance

We investigated the function of nuchal humps in second‐generation laboratory‐reared (F2) Midas cichlid individuals obtained from one breeding pair of a cross between a species with relatively small humps, *Amphilophus labiatus* (male from Lake Masaya), and a species with exaggerated humps, *Amphilophus astorquii* (female from Lake Apoyo). We chose this cross for two reasons. First, variation in hump size nicely segregated in the F2 generation. Second, as this cross was originally established for a QTL study, we had a large number of adult fish available for this cross raised under controlled density (*n* ≈ 70 adult males), which is difficult given logistical challenges imposed by rearing these large and aggressive fish to adulthood. However, we also want to acknowledge the drawback of using hybrid fish. Preference of hybrid females could differ from preference in the parental species (e.g., being transgressive). We only have anecdotal evidence for female preference in the parental species, and it will be important to explore this in more detail in subsequent studies. However, based on personal observations and previously published work (Barlow & Siri, 1997), females of the already tested species within the Midas cichlid complex have a strong preference for large humped males. Furthermore, it is worth noticing that males of some Midas cichlid species exhibit smaller humps in nature than others and it will be interesting to explore if this is associated with the ecology of the respective fish (e.g., limnetic Midas cichlid species tend to have smaller humps, personal observations).

Fish were reared in mixed‐sex groups in 760‐liter tanks (200 × 95 × 40 cm) and fed twice a day with red mosquito larvae and fish pellets. Water temperature was maintained at 28 ± 1°C, and artificial lighting was provided in a 12:12‐h light–dark cycle. Feeding, water temperature, and illumination were not altered during the experimental trials. Three months prior to the start of our experiments, fish were sexed and separated into same‐sex group tanks.

### 2.2 Characterization and quantification of the nuchal hump

Lateral photographs of the left side of all fish were taken using a Panasonic LUMIX DMC‐FZ62 camera. Specimens were illuminated using two fluorescent lamps positioned 30 cm above the specimen. As a size standard, a ruler was positioned close to the fish in all photographs. Using ImageJ (version 1.51; Schneider et al., 2012), body size was determined by measuring standard body length in reference to the size standard and hump size was quantified by measuring hump angle (see Figure 1b for details of measurements). However, as hump size could be affected by changes in body size, we also determined two other measurements for hump size corrected for the size of the fish (see Figure 1b): corrected maximum hump height (maximum hump height corrected by standard body length) estimated as:

$$\text{corrected maximum hump height}=\frac{\text{maximum hump height}}{\text{standard body length}},$$

as well as corrected hump area (hump area corrected by head area) estimated as:

$$\text{corrected hump area}\;=\frac{\text{hump area}}{\text{head area}}.$$

All measurements were taken in triplicate from the same photograph to estimate repeatability and to control for measurement error. All analyses presented in this study were performed in *R* (R Core Team, 2021). Repeatability for standard length, hump angle, maximum hump height, hump area, and head area measurements was determined by computing intraclass correlation coefficient (*ICC*) estimates using the package *ICC* (Wolak et al., 2012). Measurements for size determination (*ICC* = 0.965) and quantification of hump angle (*ICC* = 0.996), maximum hump height (*ICC* = 0.997), hump area (*ICC* = 1.0), and head area (*ICC* = 0.997) showed high repeatability.

In order to explore a potential relationship between the three measurements for hump size and between hump size and body size, we measured 70 males and 54 females. To minimize effects of breeding cycles on hump size, sexes were kept in separate tanks and were not in breeding condition. Relationships between the measurement hump angel and the two size corrected measurements for hump size (i.e., corrected maximum hump height and corrected hump area) were assessed using Pearson's correlation coefficient. Hump angle was strongly, positively correlated with both, corrected maximum hump height (*r* = 0.923, *t* = 26.449, *df* = 122, *p *< .001) and corrected hump area (*r* = 0.915, *t *= 25.068 *df* = 122, *p *< .001). Therefore, from now on, hump angle will be used as proxy for hump size, in agreement with other studies examining nuchal humps in fish (Bleick, 1975; Liu & Sadovy de Mitcheson, 2011). Sexual dimorphism in hump size was tested using an ANOVA (Type I). Log‐transformed hump size was included as response variable, and as explanatory variables, we included log‐transformed body size, sex, and their interaction.

### 2.3 Intersexual selection—humps as ornaments

Mate‐choice experiments were conducted in large 2000‐liter tanks (170 × 160 × 80 cm, tanks were not filled to maximum capacity), water temperature was maintained at 28°C (±1°C) and the experimental tanks were divided into three compartments: one large compartment (170 × 80 × 80 cm) and two small ones (85 × 80 × 80 cm; Figure 1c). The large compartment was connected to each of the small compartments by a convex gate, the size of which could be adjusted to allow the selective passage of the smaller females but restricting that of large males (Figure 1c). Because in our experimental setup, males could not see each other, female choice was most likely based only on morphological differences and potentially behavioral differences and not on the outcome of intrasexual aggression. Mate choice was tested in 20 different females exposed to 20 unique male pairs (i.e., no female or male was tested repeatedly). A female was introduced into the experimental tank and allowed to explore the entire setup for 12 hours. After this acclimation period, two males were added to the tank, each confined to one of the small compartments. Males for each trial were closely size‐matched, the smaller male was on average only 4.04% ±3.13 (mean difference ± standard deviation of difference, *n *= 20 pairs) smaller than the larger male. In contrast, differences in hump size among males within one trial were pronounced. The males with the smaller hump sizes had on average 21.98% ±8.77 (mean difference ± standard deviation of difference, *n *= 20 pairs) smaller humps than the males with the larger hump sizes. Males were randomly assigned to either of the two compartments. The female was allowed to choose between the two males by entering and leaving their respective compartments. The position of the female was monitored three times daily (approximately every 4 h at 9 am, 1 pm, and 5 pm), and females were assumed to have chosen a mate if they stayed for two consecutive days with the same male (note that the female had the option of not choosing either male by remaining in the large compartment; however, all tested females chose one of the two males). On the second evening, the female was removed from the selected male and moved back into the large compartment to verify her choice once more. In all trials, the female went back to the same male's compartment from which she had been removed, affirming her choice. The next morning, the final position of the female was recorded and subsequently used for the analysis of female choice. In most cases, eggs were laid within this period (total duration of the experiment was 3 days) on flowerpots in the male's compartment. The experiment was terminated after 20 trials. Lateral photographs of the left side of all studied individuals were taken before the experiment, and hump size and body size were measured.

We analyzed female choice using a generalized linear model (glm) with binomial error distribution. Each trial was used as one data point and scored from the perspective of the male with the larger hump. Outcome (binary: male with larger hump was selected or rejected by the focal female) was included as response variable. Accordingly, significant deviations of the intercept from zero would indicate an effect of hump size on female choice. As explanatory variables, we included Δsize (continuous: body size male with larger hump – body size male with smaller hump) and compartment side (binary: male with larger hump was in left or right compartment). For model selection, we compared Akaike information criterion (AIC) scores for four different models (Table 1). Results for female choice are reported using the model with the lowest AIC score (outcome ~compartment side).

**TABLE 1:** Akaike information criterion (AIC) scores were used for model selection throughout experiments conducted within this study. For all experiments, the model with the lowest AIC score (ΔAIC = 0) was chosen. Model assumptions and fit were checked by visual assessment of the residuals and the binomial model testing for intersexual selection was tested for overdispersion. In‐depth explanation of the different variables used is provided in the respective paragraph in the Materials and Methods section

Experiment	Response variable	Model	AIC	ΔAIC
Intersexual selection	Outcome	Δbody size + side	25.68	1.69
		Δbody size	26.14	2.18
		side	23.96	0
		null (only intercept)	24.49	0.53
Intrasexual selection	Deviation hump size successful male	deviation hump size mating partner + newcomer	115.88	1.84
		deviation hump size mating partner	114.04	0
		newcomer	117.87	3.83
		null (only intercept)	116.40	2.36
	Deviation body size successful male	deviation body size mating partner + newcomer	56.74	2
		deviation body size mating partner	55.59	0.85
		newcomer	55.75	1.01
		null (only intercept)	54.74	0
Swimming performance	Log number of finbeats	body size * hump size	−20.68	1.67
		body size + hump size	−22.35	0
		body size	−18.13	4.22
		hump size	−19.38	2.97
		null (only intercept)	−19.28	3.07
	U_crit_	body size * hump size	414.77	0
		body size + hump size	423.24	8.47
		body size	422.95	8.18
		hump size	421.30	6.53
		null (only intercept)	422.24	7.47

### 2.4 Intrasexual selection—humps as armaments

In this experiment, we explored the ability of males to acquire and maintain a breeding territory in a crowded group tank with only two of such territories (constituted by a flower pot for spawning). We asked whether the hump size of males that succeeded in acquiring a territory differed from that of non‐successful males. Midas cichlids are highly territorial fish. Only the most dominant males are able to acquire and maintain a territory. Even if a female chooses a specific male, couples will be able to spawn only if the male can defend the territory from intruders (McKaye, 1984). Therefore, although we are aware of the contribution of female choice in this experiment, successful spawning events can also be attributed to the males’ ability of defending a territory. Prior to the experiment, hump size and body size of all fish were determined. Males and females were assigned to each trial as to maximize variation in hump size between individuals in the particular trial. Within the experiment, male hump sizes ranged from 9.6° to 26.4° (Figure 1a). Differences in female hump sizes ranged from 10.5° to 18.6°. To minimize the effect of body size on mate choice, particularly large or small individuals were excluded from the experiment, resulting in a range of body size from 25.01 cm to 28.54 cm in males and from 17.26 cm to 21.94 cm in females. We conducted 21 trials, each with eight sexually mature males and four mature females present. All fish were introduced into a 2000‐liter experimental setup (170 × 160 × 80 cm) in which water temperature was maintained at 28°C (±1°C). The experimental tank was divided into three compartments: one large compartment (170 × 80 × 80 cm) and two small compartments (85 × 80 × 80 cm), each with a flower pot and separable from the large compartments by controllable gates (Figure 1d). Successful males were defined as those that acquired a territory, initiated courtship behavior, attracted a female to their territory, and proceeded to egg laying. The duration from territory acquisition to egg laying was highly variable and ranged from few days to a few weeks. During that time, the experiment was visually inspected two times daily (morning and late afternoon) for the presence of eggs. Following egg laying, the spawning couple was trapped by releasing a gate that closed off the small compartment (Figure 1d). The couple was then removed from the setup and replaced by a randomly selected (in regard to hump and body size) new male and female. We are aware that replacing only dominant fish can affect the experiment. However, given the appropriate group size per trial to maintain a crowded environment, using each fish only once would have required a large number of experimental animals. This would have neither been possible ethically nor logistically since Midas cichlids are very large (e.g., >20 cm for males) and require a lot of space for rearing and maintenance. Additionally, males are highly aggressive and rearing a large number of them becomes logistically challenging given the difficulties due to aggression of keeping them in common tanks. We analyzed both, the influence of male hump size and body size independently, using two linear models (lm). For each successful spawning male, the deviation in hump size and body size from the male group mean within the trial was calculated (male with the largest hump or body size will have a value >0 and <0 for male with the smallest hump or body size) and used as response variable. Accordingly, significant deviations of the intercept from zero would indicate an effect of hump size or body size of the male on pair formation. As explanatory variables, we included the trait value of the female mating partner (deviation in hump size or body size from the female group mean) and we corrected for a potential newcomer effect (i.e., male newly added to the experiment having an advantage over resident males inferior in the previous round; newcomer: binary: male was newly introduced to the setup or already present in the previous trial). For model selection, we compared Akaike information criterion (AIC) scores of four different models (Table 1). Results are reported using the model with the lowest AIC score for hump size (deviation hump size male ~deviation hump size mating partner) and body size (deviation body size male ~null (only intercept)).

### 2.5 Swimming performance—energetic costs and endurance

Swimming performance was measured in a 185‐liter swim tunnel (Loligo ^®^ Systems, Denmark). Flow speeds in the tunnel (10–225 cm s^−1^) were generated by a motor (Loligo ^®^ Systems, Denmark). Water temperature was 25.5 ± 0.5°C. A mirror adjusted at a 60° angle above the swim tunnel allowed dorsal filming of the fish. All trials were recorded using a video camera (Panasonic Full HD; HC‐V110). The swimming performance of 51 males was measured in three consecutive phases (Figure 1e). Fin beats could not be accurately measured for one male that was subsequently excluded. *Phase I* consisted of a 15‐minute acclimation period with no flow in the tunnel. In *phase II*, we investigated if males with large nuchal humps incurred higher energetic costs during swimming due to potential effects of the hump on the hydrodynamics of the fish. As proxy for energetic costs, we used the number of pectoral fin beats per period of time (Tudorache et al., 2009). Fish were allowed to swim at an initial flow speed of 15 cm s^−1^ for 10 min. Flow speed was then increased in 15 cm s^−1^ increments every 10 min to a final velocity of 60 cm s^−1^. Total number of fin beats (left + right pectoral fin) was manually counted over a period of 93 s at 60 cm s^−1^. Subsequently, the number of fin beats was log‐transformed to achieve a normal distribution. We analyzed fin beats using an ANOVA (Type I). Standard length, hump angle, and their interaction were treated as predictor variables, and the log transformed number of fin beats was included as the response variable. Model selection was based on AIC scores (Table 1).

During *phase III*, we determined critical sustained swimming speed (U_crit_, Brett, 1964). Flow speed started at 62.5 cm s^−1^, and velocity was increased in 2.5 cm s^−1^ increments every 5 min until the fish was exhausted. The experiment was terminated when the fish drifted back onto the grid of the swim tunnel and remained there for 1 min. Our experiments might not provide a comparable measure of U_crit_ to studies using riverine species such as trout (Beamish et al., 1989), due to the use of shorter incline steps compared to those studies and due to differences in velocity inclines between *phase II* and *phase III*. However, as we were interested only in relative performance of fish with varying hump sizes, our results provide a useful proxy for individual endurance. After exhaustion, fish were removed from the swim tunnel, photographs of the fish were taken, and hump size and body size were determined. Critical swimming speed was estimated as U_crit_ according to an equation from Brett (1964):

$$U_{\text{crit}}=u_{i}+\left(\frac{t_{i}}{t_{\mathrm{ii}}}u_{\mathrm{ii}}\right)$$

with *u_i_* being the highest velocity increment that could be maintained for the entire 5 min time interval, *t_i_* the time interval spent at the maximum exhaustion velocity, *t_ii_* the time interval of each increment (5 min), and *u_ii_* the velocity increments (2.5 cm s^−1^). U_crit_ was analyzed using an ANOVA (Type I) with standard length, hump angle, and their interaction as predictor variables and U_crit_ as response variable (see Table 1 for model selection). Interactions were explored using the package *interactions* (Long, 2019).

To test for repeatability of fin beats, U_crit_ and hump angle, swimming performance was tested twice for 14 of the 51 males. The second trial was conducted 2–4 weeks after all initial experiments were finished, and intraclass correlation coefficient (*ICC*) estimates were calculated as a measurement of repeatability using the package *ICC* (Wolak et al., 2012). Hump angles were repeatable between the trials (*ICC* = 0.889). Also, the repeatability of U_crit_ was remarkably high (*ICC* > 0.9). Repeatability for pectoral fin beats was intermediate (*ICC* = 0.631), but given that we are quantifying a behavioral measure, we consider this an acceptable repeatability.

## 3 RESULTS

### 3.1 Characterization and quantification of the nuchal hump

**FIGURE 2:**
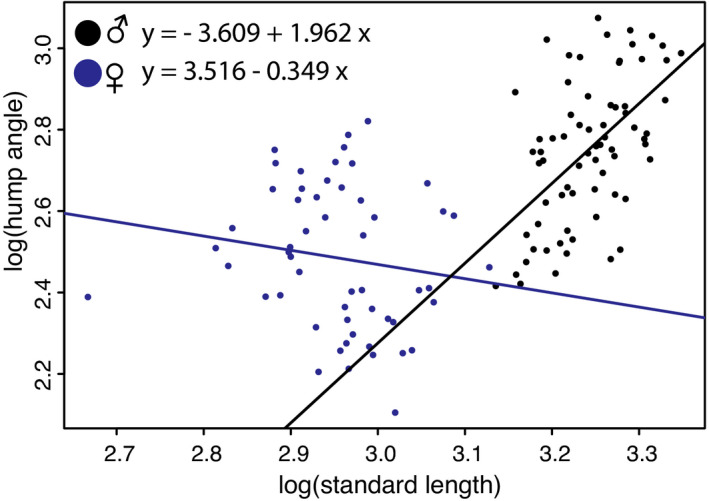
Relationship between log(hump angle) and log(standard length) and their regression lines for males (black) and females (blue). In males, hump size increased with increasing body size. In contrast, in females, hump size did not increase with increasing body size

As expected based on its proposed role in mating, sexes differed in their hump size. In males, hump angles ranged from 11.2° to 21.6°, whereas in females, they ranged only from 8.2° to 16.8°. Hump size was significantly affected by differences in body size (Table 2), with larger individuals exhibiting bigger humps. However, as body size strongly differed between the sexes, the variables body size and sex were highly correlated, explaining the only marginally significant effect of sex on hump size (Table 2). The significant interaction between body size and sex (Table 2) indicates that while with increasing body size, males grew disproportionally large humps, female hump size was not affected by an increase in body size (Figure 2).

**TABLE 2:** Results of ANOVA using log(standard length), sex and their interaction as explanatory variables and log(hump size) as response variable

	log(hump angle)		
	*df*	*F*‐value	*p*‐value
log(standard length)	1	81.784	<.001
sex	1	3.637	.059
log(standard length) * sex	1	22.117	<.001
residuals	120		

### 3.2 Intersexual interaction—humps as ornaments

When given the choice between two males matched for body size but differing in the size of their nuchal humps, females selected males with the larger hump more often than expected by chance, as indicated by the significant, positive deviation of the intercept from zero (*estimate* = 2.197, *z* = 2.085, *p* = .037, *df* = 19). Only 5 out of 20 females formed a couple with males with the smaller hump. Compartment side of the focal male had no effect on female choice (*estimate* = −1.792, *z* = −1.450, *p* = .147, *df* = 18).

### 3.3 Intrasexual interaction—humps as armaments

**FIGURE 3:**
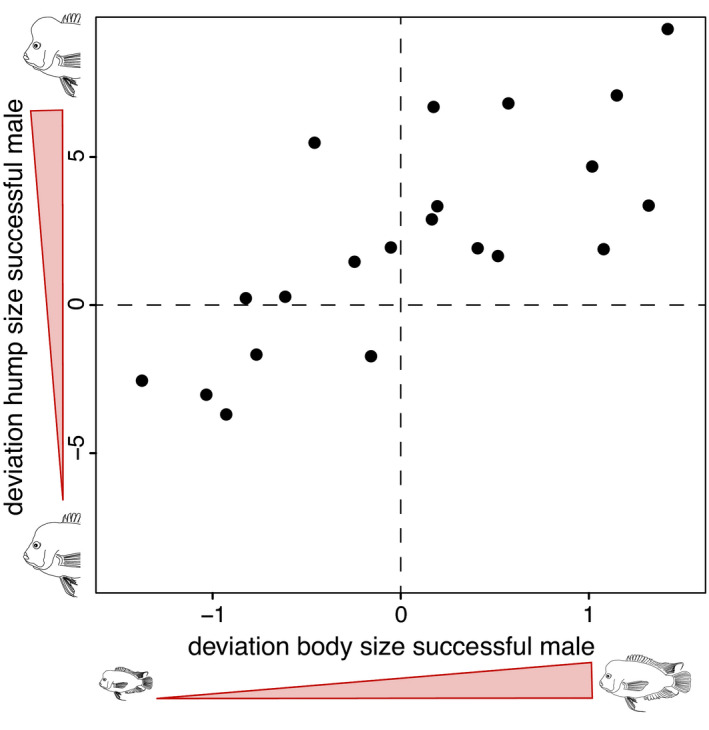
Deviation of successful males (i.e., males that successfully acquired and maintained a territory) in hump size and body size from same sex group mean. Males with larger hump size were more successful in pair formation. In contrast, body size did not affect success of males

Aggressive encounters between males competing for territories corresponded to earlier descriptions in the literature reporting aggression in male Midas cichlids (Barlow et al., 1986). More specifically, as described by Barlow et al. (1986), males approached each other flaring their opercula until fights escalated in biting and eventually jaw‐locking of the opponent. Although we did not formally test this, we assume that hump size was used to intimidate opponents and could have been potentially beneficial during jaw‐locking. Males with large humps (positive deviation from the same‐sex group mean) acquired and maintained a territory more often than expected by chance (Figure 3), indicated by the significant, positive deviation of the intercept from zero (*estimate* = 2.144, *t* = 2.95, *p* = .008, *df* = 19). The larger the male hump, the smaller was the hump of its female mating partner (*estimate* = −0.777, *t* = −2.093, *p* = .050, *df* = 19). In contrast, male body size did not influence pair formation (*estimate* = 0.074, *t *= 0.409, *p* = .687, *df* = 20; Figure 3).

### 3.4 Swimming performance—energetic costs and endurance

**FIGURE 4:**
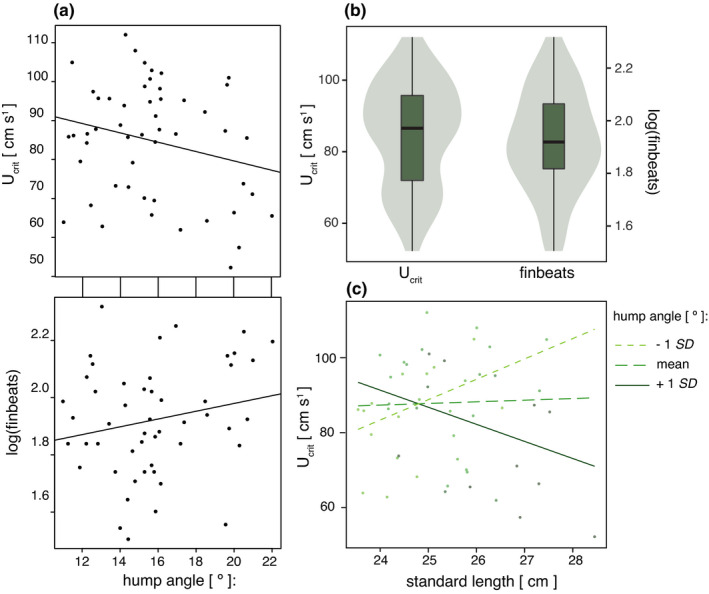
Influence of hump size on swimming performance. (a) Correlation between U_crit_ or log(finbeats) and hump size. (b) Range and distribution of the variables U_crit_ and log(finbeats). (c) Interaction between standard length and hump angle explains differences in U_crit_ between male Midas cichlids. As hump reference, −1 SD, mean and +1 SD values of hump angle were used. Differences in hump size among small males had only minor effects on their endurance. In contrast, large males with large humps were exhausted more quickly than equally large males with small humps

Energetic costs imposed on fish during swimming were estimated by the number of pectoral fin beats of the tested individuals (Figure 4b). Fish with larger humps used their pectoral fins more often than their conspecifics with smaller humps (Table 3; Figure 4a). Standard length had no significant effect on the number of fin beats.

**TABLE 3:** Results of ANOVA using standard length and hump angle as explanatory variables and log number of fin beats as response variable

	log(pectoral fin beats)		
	*df*	*F*‐value	*p*‐value
standard length	1	0.918	.343
hump angle	1	6.226	.016
residuals	47		

In an effort to analyze how hump size affects endurance, we determined the critical swimming speed (U_crit_, Figure 4b). Males showed high variation in U_crit_, with some failing to maintain performance at every velocity incline from 52.25 cm s^−1^ to 112.05 cm s^−1^. When considering which factors determined individual U_crit_, we found a significant effect of the interaction between body size and hump size (Table 4). Although in smaller fish, the influence of hump size on U_crit_ was negligible, larger fish with larger humps were exhausted more quickly than equally sized fish with smaller humps (Figure 4c).

**TABLE 4:** Results of ANOVA using standard length, hump angle, and their interaction as explanatory variables and U_crit_ as response variable

	U_crit_		
	*df*	*F*‐value	*p*‐value
standard length	1	1.523	.223
hump angle	1	1.976	.166
standard length * hump angle	1	10.703	.002
residuals	47		

## 4 DISCUSSION

Exaggerated traits evolve most commonly through sexual selection and often act as ornaments in mate choice and/or armaments in rivaling behavior (Andersson, 1994; Darwin, 1871). In cichlid fishes, research on exaggerated traits has focused mainly on their remarkable coloration phenotypes (e.g., Kratochwil et al., 2018; Seehausen & van Alphen, 1998; Sefc et al., 2014; Torres‐Dowdall et al., 2014), whereas little attention has been paid to another striking morphological feature exhibited by multiple cichlid species: the nuchal hump (Barlow & Siri, 1997; Bleick, 1975; Lecaudey et al., 2019; Meek, 1904, 1907; Takahashi, 2018). Here, our results suggest that nuchal humps could function both as sexually selected ornaments and armaments in the Midas cichlid species complex. Furthermore, we show that nuchal humps are associated with costs in terms of swimming ability, through which natural selection may counteract the further growth of this trait.

Highly exaggerated traits have been shown to serve important signaling functions as ornaments during intersexual selection (Andersson, 1986, 1994; Darwin, 1871). In mate choice, females often select males bearing the most exaggerated character states of such ornaments (Andersson, 1982a; Morehouse & Rutowski, 2010). Conforming this, we found a clear preference in female Midas cichlids for males with larger hump size among size‐controlled males. This preference indicates that nuchal humps indeed represent a sexually selected ornament. This interpretation rests on the assumption that females of the hybrid cross used in this experiment do not show transgressive preferences for hump size compared to the parental species (see Materials and Methods). However, our knowledge of the system and previously published studies (e.g., Barlow & Siri, 1997) suggest that female preference for large‐humped males is strong in already tested species within the Midas cichlid complex. As a nuchal hump significantly affects the appearance of a fish, other individuals’ perception of the bearers’ body size is likely altered. Strong female preference for large males has already been highlighted in cichlids, and it was argued that body size can even serve as proxy for male spawning experience (Beeching, 1992; Noonan, 1983; Perrone, 1978). Interestingly, the effect of large hump size might even outweigh small differences in body size, as our results showed no effect of male standard length in contrast to a significant effect of hump size when females chose among potential mates. Assuming that nuchal humps strongly alter how their bearers’ size is perceived, they could not only be important for mate choice but might also play an important role in male–male conflicts, as body size can also be an indicator for male aggressiveness and the ability to maintain territories (Barlow, 1991, 1998).

Intrasexual interactions are often competitive and lead to differential success in obtaining a specific resource (such as territories or mates; West‐Eberhard, 1984). During such rivaling intraspecific aggressive encounters, exaggerated traits can constitute armaments that can act as weapons used in combat, and/or signals that are used to threaten rivals or have an intermediate function on the so‐called weapon–signal continuum (McCullough et al., 2016). Among Midas cichlids, competition for territories or defense of broods is highly aggressive (Earley et al., 2006). However, this aggression differs among the sexes, typically males are more aggressive especially in the early phases of reproduction (coinciding with the most exaggerated state of the hump) (Holder et al., 1991). This aggression often climaxes in male–male jaw‐locking combat, during which rivals grab each other's mouth (Rogers, 1995; Wazlavek & Figler, 1989), often rapidly and vigorously turning sideways in an attempt to tear apart their opponent's lip. When such encounters occur in confined environments, such as an aquarium, they often result in severe injuries or even the death of the subordinate male, if it is not removed from the setup. Due to the aggressive nature of these interactions, body size has been claimed to play a crucial role in signaling dominance to rivals (Neat et al., 1998; Odreitz & Sefc, 2015). As we assumed that nuchal humps alter how their bearers’ body size is perceived, we tested if they constituted an armament in same‐sex competition for breeding territory acquisition. Our results are in line with this assumption (Figure 3). Males with larger humps acquired breeding territories more often than expected by chance (Figure 3). Nuchal humps could provide an important advantage in combat for breeding territories as they might increase leverage to injure the rival during jaw‐locking. However, even if nuchal humps are not being directly beneficial in head‐to‐head fights among males (Barlow & Siri, 1997), they might likely prevent fighting between hierarchically uneven rivals in the first place, as has been demonstrated for differences in body size (Wazlavek & Figler, 1989). For instance, an increase in body volume through the nuchal hump can alter its bearer's near‐flow water movement (McHenry & Liao, 2014). This can be detected by potential rivals through their mechanosensory lateral line system and used as an important source for the non‐visual assessment of the opponents fighting ability (Butler & Maruska, 2015). Therefore, nuchal humps might represent an intermediate stage in the weapon–signal continuum, by functioning both as a armament and a signal for fighting ability, posing a threat to potential rivals (McCullough et al., 2016). Humps functioning along the weapon–signal continuum provides an alternative to the hypothesis of Barlow and Siri (1997) that the nuchal hump influences sex recognition. After showing that male Midas cichlids mainly approached humpless dummies when presented with models with varying hump sizes, Barlow and Siri concluded that nuchal humps mainly facilitate sex recognition. However, more modest hump sizes might not only indicate being female but could also signal the presence of an inferior male that can be defeated. In contrast, dummies with large humps were not approached, perhaps because the humps signal dominance. Of course, male dominance represents a highly complex phenotype that might depend on various aspects. Some of them, such as hump size, might be more conspicuous and easier to test than others. Future studies exploring the contribution and interplay of different factors contributing to dominance of male Midas cichlids will be important for our understanding of their behavior. Yet, even if correlated with other traits, large hump size likely constitutes a sexually selected armament that intimidates rivals and thereby contributes to the acquisition of breeding territories.

Taken together, these results suggest that the nuchal hump might be a signal of dual function in cichlid communication acting as an ornament in female choice and as armament in male–male conflicts. Given these advantages, the question arises: Why does sexual selection not favor the continuous growth of nuchal humps? One potential explanation for the absence of continuous trait increase by directional sexual selection is generally provided by Fisher's runaway selection theory that predicts that natural selection will counteract further increase of exaggerated traits when they become too costly (Baldauf et al., 2010; Fisher, 1930; Heinen‐Kay et al., 2014; Stuart‐Fox & Ord, 2004). Exaggerated secondary sexual characteristics are often costly to produce and to maintain (Møller, 1996). At the same time, this costliness has been used as the basis for handicap models that explain how exaggerated traits can be an honest signal indicating their bearers’ condition (Andersson, 1986; Cotton et al., 2004; Iwasa et al., 1991; Johnstone, 1995; Pomiankowski, 1987; Zahavi, 1975). The costs of nuchal humps in terms of swimming performance of cichlid fishes have only rarely been studied (but see Raffini et al., 2020). However, results obtained from other fish suggest that humps can negatively influence swimming performance through increasing drag and expanding energetic costs and that they require extra effort in locomotion and position holding (Portz & Tyus, 2004). Our swimming performance experiments show that nuchal hump size also imposes costs on male Midas cichlids by negatively affecting swimming performance. As hump size increases, males use their pectoral fins more often, most likely to compensate for the loss of maneuverability due to the increased drag imposed by the hump (Table 3), as reported to be provided by paired fin movements in other species (Sfakiotakis et al., 1999). This elevated number of pectoral fin beats increases the energetic demand and might hasten exhaustion (Table 4). Decreased endurance in males with large humps was especially apparent in large‐sized males, which are the ones most likely to reproduce in nature (Rogers & Barlow, 1991; Figure 4). Energy expenditure *per se* might translate into evolutionarily significant fitness costs both in terms of mortality and reproductive success (Kotiaho, 2000). In Midas cichlids, negative effects on reproductive success are highly probable considering the biological relevance of swimming performance for males during territory and brood defense (McKaye & Barlow, 1976). During territorial acquisition and spawning, males must respond fast to intruders (Rogers, 1988), and the ability to direct locomotion precisely is important for succeeding in male–male competition. It requires endurance to repeatedly deter competitors and potential egg and larvae predators (McKaye & Barlow, 1976; Rogers & Barlow, 1991). Consequently, our assessment of swimming performance in Midas cichlids illustrates that nuchal humps might be associated with non‐negligible fitness costs for their bearers. This likely results in natural selection capping further trait exaggeration, as it would be expected under the model of runaway sexual selection (Fisher, 1930). Alternatively, the gain in benefits of increasing hump size might gradually decrease, as exceptionally large humps might be associated with mechanical constraints (e.g., paradox of the weakening combatant; O’Brien & Boisseau, 2018). Although mechanical constraints might contribute to restricting hump growth, the observed costs of having larger humps in regard to swimming performance suggest that natural selection is at play.

Exaggerated traits, widespread across different taxa, are often a product of sexual selection (e.g., Andersson, 1982a; Emlen et al., 2006; Malo et al., 2005; Petrie & Williams, 1993). Although nuchal humps are a peculiar morphological phenotype of cichlid fishes, their function in sexual selection has not been investigated thoroughly (but see Barlow & Siri, 1997). In this study, we find that nuchal humps likely constitute a trait with dual functions: an ornament preferred by females and an armament used in intrasexual competition. Although it is commonly assumed that such exaggerated traits are costly (Andersson, 1994; Emlen, 2001; Fitzpatrick et al., 1995; Møller, 1996), experiments exploring potential costs are rarely conducted (e.g., Barbosa & Møller, 1999; Somjee et al., 2018; Zhang et al., 2016). Our results highlight the costly nature of nuchal humps by showing their negative effects on locomotive ability. Even though further experiments will be required to quantify the impact of this effect on fitness, based on the breeding biology of Midas cichlids, we can infer that humps might affect reproductive success. Therefore, a trade‐off between sexual and natural selection is likely responsible for shaping hump sizes in cichlids.

Under which evolutionary scenario this conspicuous trait evolved is beyond the scope of this study and remains an open question. Exploring prerequisites of different sexual selection models (reviewed in Kokko et al., 2006), such as condition dependence, heritability, and direct and/or indirect benefits, will provide further answers. This study adds to the few examples that experimentally investigated the function and costs of exaggerated traits in the context of displays, rivalries, and performance, contributing to a more complete understanding of extravagant male traits.

## CONFLICT OF INTEREST

None declared.

## AUTHOR CONTRIBUTIONS

**Sina Julia Rometsch:** Conceptualization (equal); Data curation (lead); Formal analysis (lead); Funding acquisition (supporting); Methodology (lead); Visualization (lead); Writing‐original draft (lead); Writing‐review & editing (lead). **Julian Torres Dowdall:** Conceptualization (equal); Formal analysis (equal); Methodology (equal); Supervision (equal); Writing‐review & editing (equal). **Gonzalo Machado‐Schiaffino:** Data curation (supporting); Writing‐review & editing (supporting). **Nidal Karagic:** Data curation (supporting); Writing‐review & editing (supporting). **Axel Meyer:** Conceptualization (equal); Funding acquisition (lead); Resources (lead); Writing‐review & editing (supporting).

## Data Availability

All data are archived in DRYAD: https://doi.org/10.5061/dryad.31zcrjdn1.
